# Nomograms for Predicting Disease-Free Survival in Patients With Siewert Type II/III Adenocarcinoma of the Esophagogastric Junction Receiving Neoadjuvant Therapy and Radical Surgery

**DOI:** 10.3389/fonc.2022.908229

**Published:** 2022-06-08

**Authors:** Zhenjiang Guo, Honghai Guo, Yuan Tian, Ze Zhang, Qun Zhao

**Affiliations:** ^1^ Third Surgery Department, The Fourth Hospital of Hebei Medical University, Shijiazhuang, China; ^2^ Department of Gastrointestinal Surgery, Hengshui People’s Hospital, Hengshui, China

**Keywords:** nomogram, disease-free survival, neoadjuvant radiotherapy, prognosis, esophagogastric junction adenocarcinoma

## Abstract

**Objective:**

This study aimed to develop prognostic prediction models for patients with Siewert type II/III adenocarcinoma of the esophagogastric junction (AEG) who received neoadjuvant therapy (neoadjuvant chemoradiotherapy or neoadjuvant chemotherapy) and radical surgery. A baseline nomogram and a post-operative nomogram were constructed before neoadjuvant therapy and after surgery. The predictive performance of the constructed nomograms was internally validated and compared to the TNM staging system.

**Materials and Methods:**

A total of 245 patients diagnosed with Siewert type II/III AEG and treated with neoadjuvant therapy followed by radical surgery at The Fourth Hospital of Hebei Medical University between January 2011 and December 2017 were enrolled. The variables before neoadjuvant therapy were defined as baseline factors, while the variables of baseline factors along with the variables of treatment and postoperative pathology were defined as post-operative factors. To construct the corresponding nomograms, independent predictors of baseline and post-operative factors were identified. The C-index and a time-dependent receiver operating characteristic curve were used to evaluate the model’s discrimination ability. The calibration ability of the model was determined by comparing the probability of predicted free-recurrence to the actual free-recurrence. Decision curve analysis (DCA) was used to determine the clinical usefulness of the nomogram.

**Results:**

Among the baseline factors, age, cT stage, cN stage, Borrmann type, and staging laparoscopy were independent prognostic predictors. In contrast, among the post-operative factors, age, cN stage, staging laparoscopy, ypT stage, clinical response, number of positive lymph nodes, number of negative lymph nodes, laurén classification, and lymphatic, or perineural invasion (VELPI) were independent prognostic predictors. The two nomograms were constructed using the independent predictors of prognosis. The C-indexes for the baseline and post-operative nomograms were 0.690 (95% CI, 0.644-0.736) and 0.817 (95% CI, 0.782-0.853), respectively. The AUCs of the baseline nomogram at 3 and 5 years were both greater than cTNM (73.1 vs 58.8, 76.1 vs 55.7). Similarly, the AUCs of the post-operative nomogram were both greater than ypTNM (85.2 vs 69.1, 88.2 vs 71.3) at 3 and 5 years. The calibration curves indicated that both models had a high degree of calibration ability. By comparing the DCA at 3 and 5 years, we determined that the two nomograms constructed had better clinical utility than the TNM staging system.

**Conclusions:**

The constructed nomograms have a more accurate predictive ability than the eighth edition TNM staging system, which can be useful for treatment selection and follow-up monitoring of patients.

## Introduction

Over the last few decades, the incidence of adenocarcinoma of esophagogastric junction (AEG) has increased globally, raising new concerns (1, 2). AEG is a term that refers to digestive tract malignancy that occurs in a special anatomical site within the epicenter located 5 cm above and below the esophagogastric junction (EGJ). Siewert typing is the world’s most widely used method. Siewert type II AEGs have an epicenter located between 1 cm above and 2 cm below the EGJ, and Siewert type III AEGSs have an epicenter located 2-5 cm below the EGJ (3). In patients with Siewert type II/III AEG, surgical resection is the primary treatment. However, because the majority of patients are in the advanced stage at the time of diagnosis, the 5-year survival rate is only 20%-30% even with radical resection (4–6). Neoadjuvant chemoradiotherapy and neoadjuvant chemotherapy are the primary components of neoadjuvant therapy for AEG. Neoadjuvant therapy has been shown to improve the prognosis of AEG patients by reducing the clinical stage of the tumor and increasing the rate of radical surgical resection. According to the current study, both methods of neoadjuvant therapy appear to have comparable survival benefits (7).

The TNM staging system issued by the American Joint Committee on Cancer (AJCC) is the primary tool for determining the patients’ treatment mode and prognosis. The 8^th^ edition of the TNM staging system included clinical staging (cTNM) and post-neoadjuvant pathological staging (ypTNM), as well as the proposal to use the staging system for esophageal and gastric cancer for Siewert type II and III AEG, respectively (8). These updates provide more precise guidance for Siewert type II/III AEG patients receiving neoadjuvant therapy. However, because the TNM staging system is limited to anatomical variables such as tumor infiltration depth, lymph node invasion, and distant metastasis, the prognosis of patients with the same TNM staging remains heterogeneous. The integration of multiple factors, including patient demographic characteristics, treatment, and clinicopathological characteristics in the construction of a nomogram prediction model, has become a trend in tumor prognosis research and has been endorsed by the AJCC (9). The nomogram has been used to assist in the selection of individualized treatment and the development of follow-up strategies for patients with a variety of tumor types (10–12). Among gastrointestinal tumors, the nomogram is most widely used for colon cancer since it has been shown to be more clinically useful than the TNM staging system in predicting patient recurrence or survival (13–15). On the basis of molecular and clinicopathological features, a third-generation clinical calculator for colon cancer has been established (16). Numerous research has established nomograms for AEG. Gabriel E et al. (17) developed a prognostic calculator for patients with esophagus adenocarcinoma based on patient and treatment factors, which could predict the individual survival probability of patients receiving or not receiving neoadjuvant chemoradiotherapy before treatment. Zhou et al. (18) developed a nomogram for AEG patients using the Surveillance, Epidemiology, and End Results (SEER) database. Liu et al. (19) established Siewert II AEG patients undergoing preoperative radiotherapy based on the SEER database. Chen (20) established a nomogram for patients with Siewert type II/III AEG who did not receive preoperative treatment. Lemini R et al. (21) further externally validated several currently existing prognostic prediction models for the patients with esophageal adenocarcinoma. Unfortunately, none of these models showed satisfactory prediction accuracy. To our knowledge, we are the first to develop the prognostic prediction model for patients with Siewert type II/III AEG that includes both neoadjuvant chemoradiotherapy and neoadjuvant chemotherapy in combination with radical resection. To correspond to the TNM staging system, a baseline nomogram and a post-operative nomogram were constructed before the neoadjuvant therapy and after surgery to determine the probability of free-recurrence at 3 and 5 years, respectively. The predictive performance of the constructed nomograms was internally validated and compared to the TNM staging system.

## Materials and Methods

### Study Population

A total of 245 patients diagnosed with Siewert type II/III AEG who received neoadjuvant therapy followed by radical surgery at The Fourth Hospital of Hebei Medical University between January 2011 and December 2017 were included in the study. The following inclusion criteria were used (1): gastroscopy and histologically confirmed Siewert type II/III AEG; (2) patients treated with preoperative neoadjuvant chemoradiotherapy or neoadjuvant chemotherapy followed by R0 resection. The exclusion criterion was as follows:(1) absence of important clinicopathological or therapeutic data; (2) patients with multifocal gastric cancer or other malignancies. (3) the presence of distant metastases before neoadjuvant therapy, including patients with occult peritoneal metastases or positive laparoscopic cytology during staging laparoscopy. (4) death within 30 days after surgery. The study was reviewed and approved by the Ethics Committee of The Fourth Hospital of Hebei Medical University. The patients provided written informed consent.

### Variables

Demographic, clinicopathological, and therapeutic data were retrospectively extracted from medical records. The variables included age at diagnosis, sex, clinical stage, Borrmann type, Siewert type, pre-treatment tumor markers, type of neoadjuvant therapy, surgical procedure (subtotal/total gastrectomy), and clinical response according to the Response Evaluation Criteria in Solid Tumors (RECIST version 1.1) [complete response (CR), partial response (PR), stable disease (SD), and progressive disease (PD)], laparoscopic cytology (negative/not performed), postneoadjuvant pathologic stage, AJCC tumor regression grade, number of positive lymph nodes, number of negative lymph nodes, Laurén classification, venous, lymphatic, or perineural invasion (VELPI), Histologic grade, and HER-2 expression.

The AJCC TNM staging system, 8^th^ edition, was used in this study. In reference to previous studies, the tumor markers carcinoembryonic antigen (CEA) and carbohydrate antigen 19-9 (CA19-9) were included (22, 23). Neoadjuvant therapies included neoadjuvant chemoradiotherapy and chemotherapy. Patients receiving neoadjuvant chemotherapy were preoperatively administered with two cycles of SOX (capecitabine plus oxaliplatin) or XELOX (S1 plus oxaliplatin) regimen. Surgery was performed 4 weeks after neoadjuvant chemotherapy, while the preoperative chemotherapy regimen was repeated 4 weeks after surgery (24). Additionally, for patients undergoing neoadjuvant radiotherapy, intensity-modulated radiation therapy (IMRT) (50.4Gy/25 fraction) was used in conjunction with an XELOX chemotherapy regimen. Surgery was performed 6-8 weeks following the final dose of radiotherapy. XELOX regimen was repeated as postoperative adjuvant chemotherapy 4 weeks after surgery (25). The majority of patients scheduled for neoadjuvant therapy at our center underwent staging laparoscopy prior to treatment. Staging laparoscopy aimed to look for occult peritoneal metastases that had not been detected by imaging and to perform concurrent laparoscopic cytology. All patients underwent radical resection involving total or subtotal gastrectomy combined with D2 lymphadenectomy.

### Follow-up

Follow-up visits were conducted every 3 months for the first 2 years, every 6 months for the next 3 years, and annually thereafter. Disease-free survival (DFS) was defined as the time between the date of operation and the date of recurrence or the last follow-up if recurrence did not occur. The final follow-up was in November 2021.

### Construction and Validation of the Nomogram

The variables before neoadjuvant therapy were defined as baseline factors, whereas the variables of baseline factors along with the variables of treatment and postoperative pathology were defined as post-operative factors. All variables were subjected to univariate Cox regressions. Subsequently, Multivariate Cox analyses were conducted separately for variables with a P<0.05 in the univariate Cox regression analysis for baseline and postoperative factors. The baseline and post-operative nomograms were constructed using multivariate Cox regression based on the independent risk factors identified. Harrell’s concordance index (c-index) and a time-dependent receiver operating characteristic (ROC) curve were used to assess the model’s discrimination ability. The C-index ranges from 0.5 to 1.0, and a higher C-index indicated better discrimination of the model. The calibration ability of the model was determined by comparing the predicted probability of free-recurrence to the actual free-recurrence. Finally, decision curve analysis (DCA) was used to determine the clinical usefulness of the nomogram.

### Statistics Analysis

Categorical variables were expressed as frequency rates, while continuous variables were expressed as the median (interquartile range [IQR]). All continuous variables were subjected to linearity testing. Continuous variables with nonlinearity were modeled using restricted cubic splines. Univariate and multivariate analyses were performed using Cox regression models to determine the association between prognostic predictors and DFS, while hazard ratios (HRs) and 95% confidence intervals (CIs) were generated. To identify variables for multivariate Cox proportional hazards regression models, a backward stepwise selection method with the Akaike information criterion (AIC) was used. The nomograms were constructed based on the independent variables identified by multivariate analysis. Statistical analysis was performed using R software (version 4.0.3, http://www.R-project.org). The nomogram and calibration curve were generated using the Hmisc, rms, and ggplot2 packages, while riskRegression was used for receiver operating characteristic curve analysis (ROC), and “ggDCA” for decision curve analysis (DCA).

## Results

### Patients Characteristics and Univariate Cox Regression Analysis

**Table 1** shows the demographic, clinicopathological, and treatment data of the 245 patients included in the study. The median follow-up time was 47 (IQR, 23-82) months. The median age of the patients was 62 (IQR, 57-66) years. There were 203 (82.9%) men and 42 (17.1%) women. 71 (29.0%) cases were diagnosed as Siewert type II and 174 (71.0%) cases as Siewert type III. For clinical stages, 65 patients were classified as cT2-3 stage and 180 patients as cT4 stage. Additionally, 54 patients were classified as cN0 stage and 191 patients as a cN1-3 stage. For Borrmann type, 5 patients were type I, 86 were type II, 139 were type III, and 15 were type IV. Among the pre-treatment tumor markers, 74 patients had elevated CEA and 64 patients had elevated CA199 levels. Pre-treatment laparoscopic cytology was performed and diagnosed as negative in 184 patients, while 61 patients were not examined. Neoadjuvant chemoradiotherapy was administered to 76 patients, neoadjuvant chemotherapy with the XELOX regimen was administered to 66 patients, and neoadjuvant chemotherapy with the SOX regimen was administered to 103 patients. Total gastrectomy was performed on 168 patients, whereas subtotal gastrectomy was performed on 77 individuals. 159 (64.9%) patients received neoadjuvant therapy and achieved objective remission (CR+PR). For the post neoadjuvant pathologic stage, there were 20, 35, and 190 cases of ypT0, ypT1-2, ypT3-4, respectively. There were 125, 59, 40, and 21 cases of ypTN0, ypTN1, ypTN2, ypTN3, respectively. There were 23 cases of AJCC-TRG grade 0, 62 cases of grade 1, 101 cases of grade 2, and 59 cases of grade 3. The median number of positive lymph nodes was 0 (0, 3), while negative lymph nodes were 24 (15, 33). For the Laurén classification, 118 (48.2%) were Intestinal, 75 (30.6%) were Diffuse, and 26 (10.6%) were mixed. A total of 139 patients presented with VELPI. 188 patients showed poor or undifferentiated histological grade. 38 patients showed a positive her2 status (immunohistochemistry 3+).

**Table 1:** Demographic, treatment, and clinicopathological characteristics of patients and univariate analyses for disease-free survival.

Variable	No. (%)	Univariate analysis	
		HR (95CI)	p
**Baseline factors**			
Age, years	62 (57, 66)*	0.946 (0.918-0.975)^#^	<0.001
		1.073 (1.027-1.121)^#^	0.002
Sex			
Male	203 (82.9)	1	
Female	42 (17.1)	0.908 (0.574-1.439)	0.682
Siewert type			
II	71 (29.0)	1	
III	174 (71.0)	1.058 (0.719-1.556)	0.776
cT stage			
2-3	65 (26.5)	1	
4	180 (73.5)	2.456 (1.508-4.000)	<0.001
cN stage			
0	54 (22.0)	1	
1-3	191 (78.0)	2.374 (1.405-4.011)	0.001
Borrmann type			
I	5 (2.0)	NA	NA
II	86 (35.1)	1	0.136
III	139 (56.7)	1.369 (0.926-2.025)	0.116
IV	15 (6.1)	2.888 (1.507-5.531)	0.001
Pre-treatment CEA			
Normal (≤5 ng/mL)	164 (66.9)	1	
Elevated (> 5 ng/mL)	74 (30.2)	1.381 (0.953-2.002)	0.088
Unknown	7 (2.9)	0.958 (0.351-2.617)	0.934
Pre-treatment CA 19-9			
Normal (≤37 ng/mL)	174 (71.0)	1	
Elevated (> 37ng/mL)	64 (26.1)	1.126 (0.761-1.666)	0.553
Unknown	7 (2.9)	0.895 (0.328-2.438)	0.828
Staging laparoscopy			
Negative	184 (75.1)	1	
Not performed	61 (24.9)	1.890 (1.301-2.748)	<0.001
**Treatments**			
Neoadjuvant therapy			
nCRT	76 (31.0)	1	
nCT (XELOX)	66 (26.9)	1.260 (0.796-1.993)	0.325
nCT (SOX)	103 (42.0)	1.070 (0.702-1.632)	0.753
Gastrectomy			
Subtotal	168 (68.6)	1	
Total	77 (31.4)	1.069 (0.736-1.552)	0.726
Clinical response			
CR+PR	159 (64.9)	1	
SD+PD	86 (35.1)	2.746 (1.935-3.897)	<0.001
**Postoperative pathology**			
ypT stage			
0	20 (8.2)	1	
1-2	35 (14.3)	3.711 (0.822-16.760)	0.088
3-4	190 (77.6)	7.904 (1.952-32.010)	0.004
ypN stage			
0	125 (51.0)		
1	59 (24.1)	2.431 (1.521-3.886)	<0.001
2	40 (16.3)	5.356 (3.334-8.605)	<0.001
3	21 (8.6)	25.597 (13.805-47.461)	<0.001
AJCC-TRG			
0	23 (9.4)		
1	62 (25.3)	3.075 (0.928-10.190)	0.066
2	101 (41.2)	5.861 (1.829-18.780)	0.003
3	59 (24.1)	11.924 (3.697-38.460)	<0.001
Number of positive lymph nodes	0(0, 3)*	1.504 (1.365-1.656)^#^	<0.001
		1.121 (1.075-1.168)^#^	<0.001
Number of negative lymph nodes	24 (15, 33)*	0.983 (0.969-0.998)	0.022
Laurén classification			
Intestinal	118 (48.2)		
Diffuse	75 (30.6)	1.969 (1.338-2.899)	0.001
Mixed	26 (10.6)	1.647 (0.895-3.031)	0.109
Unknown	26 (10.6)	1.155 (0.602-2.218)	0.665
VELPI			
Absent	139 (56.7)		
Present	92 (37.6)	3.268 (2.269-4.706)	<0.001
Unknown	14 (5.7)	2.030 (0.921-4.475)	0.079
Histologic grade			
Well or moderate	57 (23.3)		
Poor or undifferentiated	188 (76.7)	1.439 (0.923-2.244)	0.109
Her-2 status			
0	58 (23.7)		
+~++	95 (38.8)	1.110 (0.696-1.770)	0.663
+++	38 (15.5)	1.986 (1.169-3.374)	0.011
Unknown	54 (22.0)	1.092 (0.647-1.845)	0.741

nCRT, neoadjuvant chemoradiotherapy; nCT, neoadjuvant chemotherapy; AJCC, American Joint Committee on Cancer; TRG, tumor regression grading; VELPI, lymphatic or perineural invasion; HR, hazard ratio; CI, confidence interval; NA, Not Available.

^*^Median (IQR); ^#^Restricted cublic spline fits for these continuous variables.

**Figure 1:**
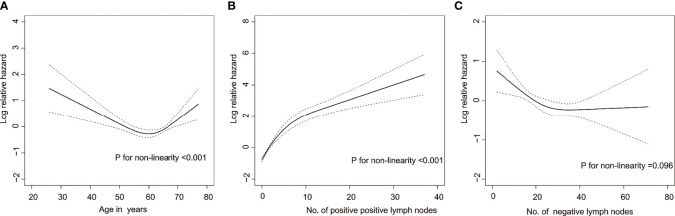
Risk of recurrence as a function of **(A)** age, **(B)** Number of positive lymph nodes, and **(C)** Number of negative lymph nodes. Solid line = risk function. Dashed lines = 95% confidence bands for the risk function.

Due to the small number of patients with Borrmann type I, these individuals were excluded from the Cox analysis. Among the included continuous variables, age and the number of positive lymph nodes had a nonlinear relationship with recurrence, but the number of negative lymph nodes had a linear relationship with recurrence (**Figure 1**). Among the baseline factors, univariate cox analysis showed that age, cT stage, cN stage, Borrmann type, and laparoscopic cytology all influenced DFS (p=<0.05). Among the treatment factors, however, the only clinical response affected DFS (p=<0.05). Among the postoperative pathological factors, ypT stage, ypN stage, AJCC-TRG, number of positive lymph nodes, number of negative lymph nodes, Laurén classification, VELPI, and Her-2 status affected prognosis (p=<0.05), as shown in **Table 1**.

### Independent Prognostic Factors Among the Baseline and Postoperative Factors

In the multivariate analysis, age, cT stage, cN stage, Borrmann type, laparoscopic cytology all had an effect on DFS among the baseline factors (**Table 2**). Among the postoperative factors, Age, cN stage, Laparoscopic cytology, ypT stage, Clinical response, number of positive lymph nodes, number of negative lymph nodes, Laurén classification, VELPI all independently influenced DFS (**Table 2**).

**Table 2:** Multivariate analyses for disease-free survival of baseline and post-therapy factors.

Variable	Multivariate analysis	
	HR (95CI)	p
**Baseline factors**		
Age (years)	0.932 (0.900-0.965)^#^	<0.001
	1.095 (1.044-1.149)^#^	<0.001
cT stage		
2-3		
4	1.638 (0.951-2.821)	0.002
cN stage		
0		
1-3	2.136 (1.241-3.674)	0.006
Borrmann type		
II		
III	1.334 (0.889-2.003)	0.164
IV	3.101 (1.596-6.026)	0.001
Staging laparoscopy		
Negative		
Not performed	2.064 (1.405-3.032)	<0.001
**Post-therapy factors**		
Age (years)	0.963 (0.928-0.998)^#^	<0.001
	1.093 (1.043-1.145)^#^	<0.001
cN stage		
0		
1-3	2.066 (1.179-3.619)	0.011
Staging laparoscopy		
Negative		
Not performed	1.550 (1.015-2.367)	0.043
ypT stage		
0		
1-2	2.734 (0.590-12.667)	0.198
3-4	4.364 (1.041-18.291)	0.044
Clinical response		
CR+PR		
SD+PD	1.496 (0.969-2.310)	0.069
Number of positive lymph nodes	1.405 (1.256-1.572)^#^	<0.001
	1.081 (1.035-1.129)^#^	<0.001
Number of negative lymph nodes	0.987 (0.972-1.002)	0.082
Laurén classification		
Intestinal		
Diffuse	1.527 (0.999-2.334)	0.050
Mixed	1.090 (0.566-2.100)	0.796
VELPI		
Absent		
Present	1.503 (0.964-2.344)	0.072

VELPI, lymphatic or perineural invasion; HR, hazard ratio; CI, confidence interval.

^#^Restricted cublic spline fits for these continuous variables.

### Construction of Baseline and Post-Operative Nomograms

**Figure 2:**
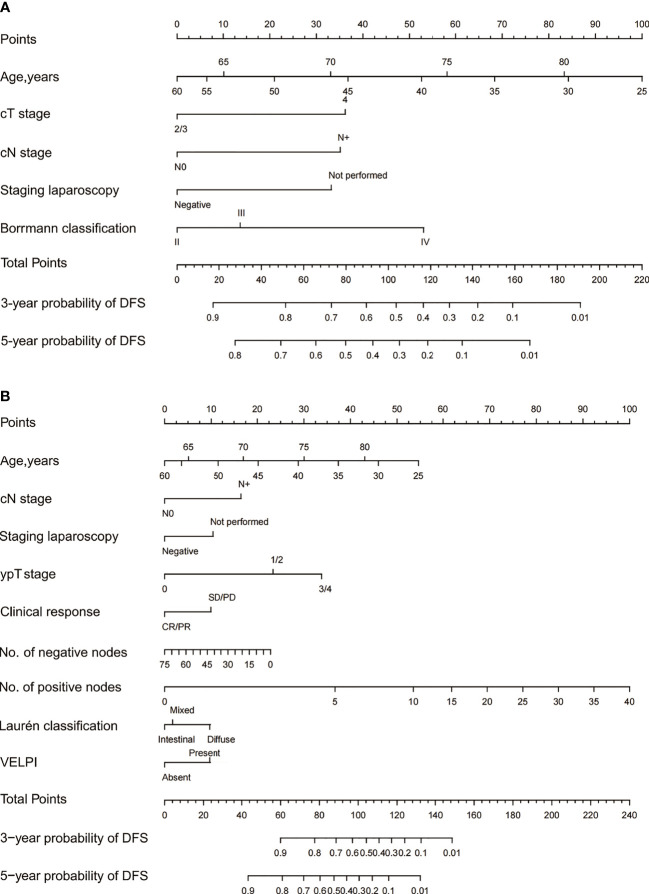
The 3- and 5-year DFS of Siewert Type II/III AEG patients were predicted by the baseline nomogram **(A)** and post-therapy nomogram **(B)**. Each variable in the model corresponded to a score, and all the scores were summed to obtain a total score corresponding to the probability of free-recurrence at 3 and 5 years.

The baseline and post-operative nomograms were constructed using independent prognostic factors identified in cox regression. Each variable in the model was assigned a score, and the scores were summed to obtain a total score corresponding to the probability of free-recurrence at 3 and 5 years. The higher the total score, the lower the probability of free-recurrence for the patient (**Figures 2A, B**).

### Internal Validation and Comparison With AJCC

**Figure 3:**
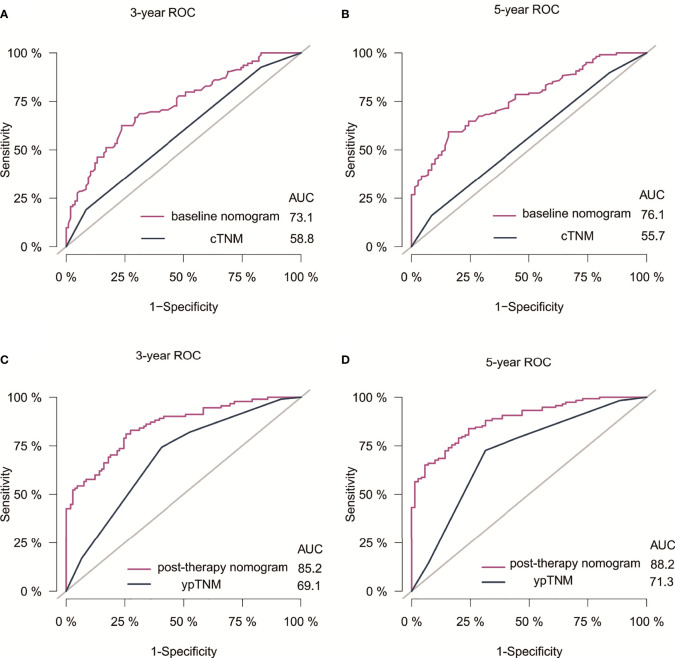
ROC of nomograms and the AJCC staging system for 3- and 5-year DFS prediction in Siewert Type II/III AEG patients. **(A)** 3-year ROC for baseline nomogram and cTNM staging. **(B)** 5-year ROC for baseline nomogram and cTNM staging. **(C)** 3-year ROC for post-therapy nomogram and ypTNM staging. **(D)** 5-year ROC for post-therapy nomogram and ypTNM staging. Receiver operating characteristic curves; AUC, Area under the curve.

**Figure 4:**
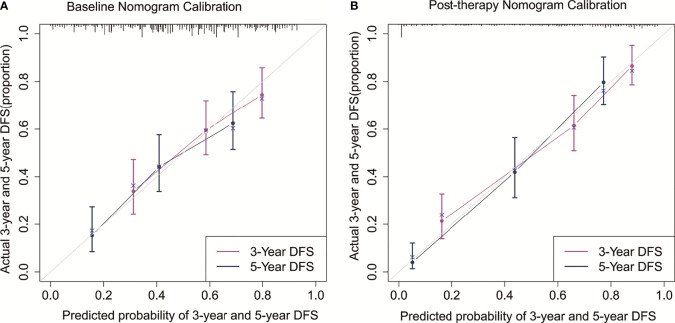
Calibration curves for the baseline nomogram **(A)** and post-therapy nomogram **(B)** predicted 3- and 5-year DFS.

**Figure 5:**
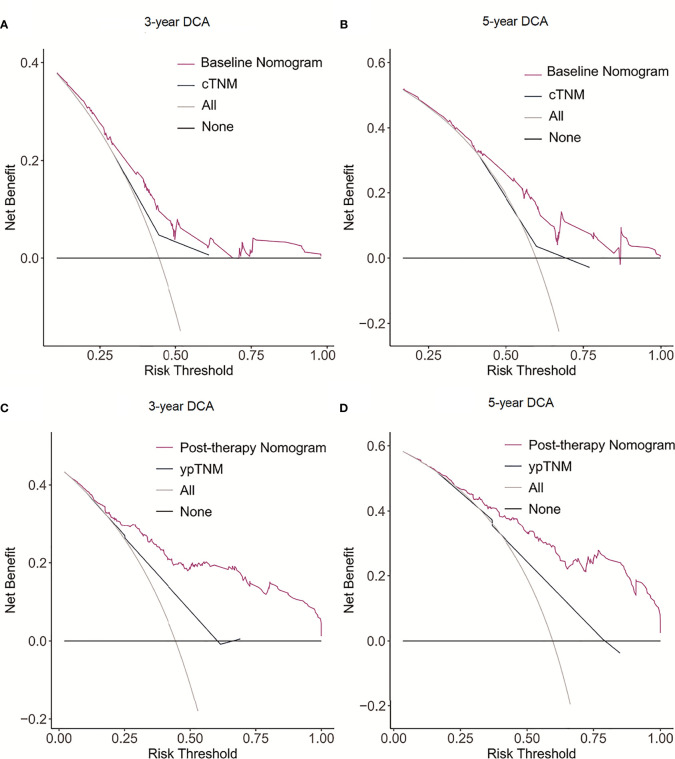
Decision curve analysis (DCA) of the nomogram model and AJCC staging model for predicting 3- and 5-year DFS. **(A)** 3-year DCA for baseline nomogram and cTNM staging. **(B)** 5-year DCA for baseline nomogram and cTNM staging. **(C)** 3-year DCA for post-therapy nomogram and ypTNM staging. **(D)** 5-year DCA for post-therapy nomogram and ypTNM staging.

The C-indexes for the model baseline and post-operative nomograms were 0.690(95% CI,0.644-0.736) and 0.817(95% CI,0.782-0.853), respectively. In the time-dependent ROC, the AUCs of the baseline nomogram at 3 and 5 years were both greater than cTNM (73.1 vs 58.8, 76.1 vs 55.7). Similarly, the AUCs of the postoperative nomogram at 3 and 5 years were both greater than ypTNM (85.2 vs 69.1, 88.2 vs 71.3), as shown in **Figure 3**. The calibration curves showed that both models had a good calibration ability (**Figure 4**). By comparing the DCA curves at 3 and 5 years, we found that the two nomograms had better clinical utility than the TNM staging system (**Figure 5**).

## Discussion

Although the optimal multidisciplinary treatment strategy for patients with advanced Siewert type II/III AEG remains controversial, neoadjuvant therapy in combination with surgical resection continues to be the primary approach. The most often used treatment modality is neoadjuvant chemotherapy or neoadjuvant radiotherapy. However, with the advent of targeted drugs and immune checkpoint inhibitors, preoperative therapies have become more diverse (26). The purpose of constructing a baseline nomogram was mainly to estimate the prognosis of individual patients before they are scheduled to receive neoadjuvant chemoradiotherapy or neoadjuvant chemotherapy and to provide a basis for an individualized therapeutic approach.

Age, cT stage, cN stage, Borrmann type, and staging laparoscopy were the predictors in the baseline nomogram. Some studies have shown that age is an independent risk factor affecting the prognosis of patients with AEG, and the prognosis worsens with increasing age (27–29). Nevertheless, other researchers have demonstrated that age is not a factor contributing to prognosis (30–32). The explanation for this variation between research could be because different studies used different subjects, treatment, and age cut-off values. In this study, age was found to be nonlinearly related to patient prognosis, with the risk of recurrence decreasing with age in patients under 60 years old and increasing with age above 60 years old. The advanced cT and cN stages were associated with a worse prognosis, which was consistent with previous studies (33, 34). The accuracy of clinical staging has significantly improved as a result of advances in imaging technologies and standardization of operations. Multi-detector computed tomography (MDCT) is the most commonly used imaging technique for the comprehensive assessment of clinical staging of AEG. MDCT has a high accuracy for cT staging of gastric cancer (35–37), but its accuracy for cN staging is relatively limited (38–40). Therefore, in this study, cN staging was divided into cN0 or cN+ to reduce bias in cN staging. Peritoneal metastasis is one of the common distant metastases sites for AEG. The presence or absence of peritoneal metastasis has a significant impact on the treatment strategy is chosen and patient prognosis (41). MDCT is currently the main method for evaluating distant metastases, with a high degree of accuracy in evaluating liver and lung metastasis. However, its assessment of peritoneal metastases is unsatisfactory, particularly for occult peritoneal metastasis. MDCT has high specificity but low sensitivity in diagnosing and evaluating peritoneal metastases (42–44). Sarela AI et al. (45) conducted a retrospective assessment of 657 patients with gastric cancer and AEG who underwent CT evaluation for M0. Peritoneal metastases were detected in 149 patients (23%) and significantly correlated with tumor location after laparoscopy, with AEG patients being more likely to have occult peritoneal metastases. In patients with progressive gastric cancer and AEG, staging laparoscopy can improve the accuracy of peritoneal metastasis diagnosis (46). In our study, 75.1% of patients underwent staging laparoscopy before neoadjuvant therapy, and the results indicated that patients who had a negative laparoscopy had a better prognosis than those who did not undergo laparoscopy. According to certain studies, diagnostic laparoscopy and cytology should be performed on all patients with Siewert II/III AEG with cT3/T4 before neoadjuvant chemotherapy to determine the presence of occult peritoneal metastases (47). Additionally, Borrmann type is a predictor in the baseline nomogram. Borrmann type I patients was excluded from this study due to the small sample size. Borrmann type III/IV patients have a worse prognosis than Borrmann type II patients, consistent with previous studies (48).

After neoadjuvant therapy and surgical resection, we added treatment and postoperative pathology variables to the baseline variables and identified independent risk factors among them to construct a post-operative nomogram. Age and laparoscopic staging were also significant predictive factors of prognosis. Among the anatomical variables of T and N elements, the number of positive lymph nodes was chosen to replace ypN staging and the number of negative lymph nodes was added to improve the predictive efficacy of the model. Additionally, the prognostic assessment of ypT staging in patients with AEG is controversial. Sisic L et al. (49) demonstrated that survival curves for patients with AEG and gastric cancer treated with neoadjuvant therapy at ypT0-2 staging were overlapping, with ypT3-4 staging showing prognostic stratification. They hypothesized that residual tumor cells following neoadjuvant therapy may remain in any layer of the GI tract, and therefore ypT staging did not provide a good stratification of prognosis. In this study, ypT staging was further optimized into ypT0, ypT1/2, and ypT3/4 categories. The results indicated that this prediction model included the four anatomical variables of T and N elements, cN staging, ypT staging, number of positive lymph nodes, and number of negative lymph nodes, but not cT staging. It has been demonstrated that the ypTNM stage rather than the initial cTNM stage is the main determinant of prognosis in neoadjuvant-treated AEG patients (50). The present findings indicate that lymph node status including the presence of preoperative lymph node metastases or more numbers of positive lymph nodes postoperatively indicated a poor prognosis, but a greater number of negative lymph nodes dissection improved prognosis. Optimized ypT staging was also found to be an independent risk factor for prognosis. In patients with AEG, clinical response was an independent risk factor for prognosis which is consistent with our previous study (24). Patients whose tumors were effectively controlled locally (CR+PR) had a better prognosis. To simplify the model, we used VELPI as a reference from a previous study on colon cancer prognosis (16). Additionally, it is an independent factor affecting prognosis in the model. Lymphovascular invasion (LVI) has been reported to be more prevalent in AEG patients than in esophageal and gastric cancers, particularly in Siewert type III. It may be associated with the development of this type of AEG in patients with chronic atrophic gastritis, a condition in which the mucosa is thin and tumor cells are more likely to invade the lymph vessels. AEGs with LVI have a worse prognosis (51). Additionally, Lauren classification is a significant independent factor affecting prognosis, with patients with a diffuse prognosis having a worse prognosis, which is consistent with previous reports (52).

The C-indexes of model baseline and post-operative nomograms are 0.690 and 0.817, respectively, indicating that both models have good predictive performance, while the postoperative nomogram model performed better. The prognosis of patients is dynamic. Both the patient’s response to neoadjuvant therapy and post-operative clinicopathological features can affect their prognosis. Therefore, the post-therapy nomogram has a better predictive performance. Additionally, the constructed nomogram had higher predictive efficiency than TNM when the AUC and DCA curves were compared. Unlike the TNM staging system, which categorizes AEG patients with Siewert type II/III into distinct staging sites, the model we constructed integrated these patients into a single model with high predictive performance. Therefore, it will be more practical and convenient for individualized patient management. Just as the clinical significance of cTNM staging and ypTNM staging in the 8th edition of the TNM staging system, we developed corresponding baseline and post-operative prediction models respectively. The models demonstrated higher prognostic predictive efficacy than the TNM staging system which can provide useful information for patient individualized treatment and follow-up.

There are some limitations to this study. First, this single-center retrospective analysis may have introduced bias in patient selection. Second, because the majority of patients with Siewert type II/III AEG are treated with general surgery in China, only patients with transabdominal radical resection were included in this study. Patients undergoing transthoracic resection were excluded due to the large variation in treatment modalities among thoracic clinicians. Third, patients with neoadjuvant targeted or immunotherapy were excluded due to the lack of sufficient follow-up time and inconsistent clinical response assessment criteria.

In conclusion, the nomogram we constructed has a more accurate predictive ability than the TNM staging system, which can provide useful information for patient treatment selection and follow-up monitoring.

## Data Availability Statement

The original contributions presented in the study are included in the article/supplementary material. Further inquiries can be directed to the corresponding author.

## Ethics Statement

The study was reviewed and approved by the Ethics Committee of The Fourth Hospital of Hebei Medical University. The patients provided written informed consent.

## Author Contributions

ZG was involved in final data analysis and manuscript writing. HG, YT, and ZZ were assisted in the data collection and analysis. QZ was involved in study design and responsible for the entire research project. All authors contributed to the article and approved the submitted version.

## Conflict of Interest

The authors declare that the research was conducted in the absence of any commercial or financial relationships that could be construed as a potential conflict of interest.

## Publisher’s Note

All claims expressed in this article are solely those of the authors and do not necessarily represent those of their affiliated organizations, or those of the publisher, the editors and the reviewers. Any product that may be evaluated in this article, or claim that may be made by its manufacturer, is not guaranteed or endorsed by the publisher.
